# How to Estimate Food Prices and Diet Costs in Population-Based Studies?

**DOI:** 10.3389/fnut.2021.728553

**Published:** 2021-10-22

**Authors:** Aline Veroneze de Mello, Flavia Mori Sarti, Regina Mara Fisberg

**Affiliations:** Department of Nutrition, School of Public Health, University of São Paulo, São Paulo, Brazil

**Keywords:** diet cost, economics, food price, linkage, population-based studies

## Abstract

Health and nutrition surveys usually comprise detailed information on health characteristics and food consumption of certain population groups; however, the lack of data collection on the food prices may pose challenges for the estimation of the diet costs for the comprehensive analysis of food demand. The Household Budget Survey (HBS) represents an opportunity to obtain the data on the food prices for the nutrition surveys in the diverse countries worldwide. Although the HBS and the health and nutrition surveys may correspond to different periods, the application of the appropriate Consumer Price Index (CPI) allows to address the changes in the relative prices to perform the linkage between the data of food consumption with information on the food prices. Therefore, the aim of this study was to present the methods for the extraction and linkage of the food prices data from the Brazilian HBS (2002–2003 and 2008–2009) by using the pairing features related to the household characteristics to match the Health Survey of São Paulo [*Inquérito de Saúde de São Paulo* (ISA-Capital)] conducted in 2003, 2008, and 2015. Data referring to the household characteristics and food prices acquired by the household members living in São Paulo municipality were selected from the HBS datasets for integration with the ISA-Capital dataset. Specific deflators referring to the food items surveyed in São Paulo were obtained from the datasets of the Brazilian Broad Consumer Price Index (BCPI). Therefore, the pairing criteria referring to time, location, and household characteristics were adopted to allow linking foods consumed by the individuals in the ISA-Capital with the prices from the foods acquired by household members interviewed in the HBS. Matching data on the key pairing criteria (location/year/household income per capita/number of residents/family profile) resulted in the linkage of 94.4% (2003), 92.6% (2008), and 81.2% of the cases (2015). Following the data linkage, it was possible to estimate diet costs per gram and per calorie including application of cooking and conversion factors. Data were presented in the International Monetary Unit under the purchasing power parity (PPP) to allow the comparison at the international level. The mean diet costs identified in the population of São Paulo municipality were $8.45 (dp = 0.38) per capita per day in 2003, $8.72 (dp = 0.24) per capita per day in 2008, and $9.62 (dp = 0.23) per capita per day in 2015. Thus, it was possible to estimate the diet costs based on the prices of food items through pairing linkage of information from the household surveys, such as the Brazilian HBS, with the health and nutrition surveys lacking information on the expenditures or prices such as the ISA-Capital. Similar procedures may be used in the diverse countries with availability of the datasets of the household expenditures and health and nutrition surveys, allowing the researchers worldwide to associate the diet quality with food demand.

## Introduction

There is substantial evidence showing that the diet costs are the key socioeconomic determinants of the food choice and consumption, particularly considering the relationship between the food prices and income of the individuals. In addition, the diet costs represent part of the causal pathway between the socioeconomic status and diet quality (1), a relationship that explains the significant inequalities in the diet quality among the different population groups (1, 2).

In addition, the assessment of the diet cost and investigation of its association with the decision-making processes involved in the food purchase are essential to design the strategies for the public policies toward the health promotion through adoption of the healthy lifestyles, especially in the low- and middle-income countries (1, 3–6). Therefore, the measurement of the diet costs comprises an important part of the analysis on the socioeconomic determinants of the dietary patterns, considering its role in the process for the adoption of the healthy food consumption patterns and fulfillment of the dietary recommendations at the population level, especially among the individuals with lower socioeconomic status (7).

Nevertheless, the lack of data collection on the food prices in health and nutrition surveys may impose barriers for the estimation of the diet costs in nutrition studies. The linkage or integration of data from independent sources comprises a technique usually adopted in the field of health information systems, performed through matching data of individuals, households, or other survey units (8, 9). In the case of nutrition surveys in Brazil, data on food prices may be obtained from the Household Budget Survey (HBS), conducted by the Brazilian Institute of Geography and Statistics (IBGE), which interview a representative sample of individuals in the Brazilian population. The HBS includes detailed data on household characteristics and food expenditures that allow the estimation of diet costs by using the prices of food items for certain periods in the different metropolitan regions of the country.

Thus, the objectives of this study were:

1. To present the method of the extraction and linkage of data on the food prices from the HBS through pairing criteria based on time, location, and household characteristics for the estimation of the diet costs in the health and nutrition surveys.
2. To demonstrate the use of the method through the example of the linkage between the data from the HBS performed in 2002–2003 and 2008–2009 into the datasets of the Inquérito de Saúde de São Paulo (ISA-Capital) conducted in 2003, 2008, and 2015.

## Materials and Methods

This study was based on the data from the HBS performed by the IBGE and the data from the health surveys conducted in the municipality of São Paulo (ISA-Capital). The HBS refers to the recurrent survey at the national level designed to identify the consumption patterns and living conditions of the Brazilian households in the different metropolitan regions of the country. The ISA-Capital comprises the health and nutrition surveys aimed at the assessment of health status, utilization of health services, social determinants of health, and lifestyle characteristics of the population living in the municipality of São Paulo.

The sample selection processes adopted in the HBS-IBGE and the ISA-Capital were based on the complex sampling design in two stages: census sectors and households. The sampling strategy adopted in the HBS-IBGE and the ISA Capital allows population-level representativeness, being based on individual and household level information, obtained through semi-structured interviews. Additional information on the surveys is available in the previous publications (10–13).

The individual- and household-level databases of the HBS were obtained from the official website of the IBGE, corresponding to information collected in the following domains:

1. Information on the sociodemographic characteristics of the household members and household characteristics
2. Information on the monetary and non-monetary earnings of the household members (≥10 years old)
3. Data on the household expenditures including the detailed information on the amount and price of foods purchased.

Data on the household expenditures from the HBS-IBGE datasets encompasses information on the acquisition of products and services during 7 consecutive days, including interviews of households with similar characteristics within the same census tracts of metropolitan regions throughout the year to allow the identification of seasonality, being especially important feature to record the differences in the food consumption and price changes according to seasons.

The pairing process was based on matching the data from the HBS-IBGE performed in 2002–2003 and 2008–2009 with the datasets of the ISA-Capital conducted in 2003, 2008, and 2015. Matching criteria included location, period, and socioeconomic and demographic characteristics of the households to link the data on the prices of foods acquired by the consumers interviewed in the HBS-IBGE with the datasets of foods consumed by the individuals interviewed in the ISA-Capital.

Therefore, the first step in matching the data was the selection of information of the households located in the urban areas of the municipality of São Paulo within the HBS-IBGE datasets, corresponding to the location of the ISA-Capital. Thus, information on state, municipality, and geographic strata were adopted in the primary filter of the dataset to comprise robust information on the food prices according to the cost of living in São Paulo municipality.

In sequence, the following additional pairing criteria referring to the period and socioeconomic and demographic characteristics of the households were selected for the linkage process:

1. Proximity of the period of the HBS-IBGE in relation to the period of the target survey ISA-Capital to identify and maintain the relative prices of foods purchased and consumed according to the economic situation of the country and the municipality:
  - HBS prices 2002–2003 → ISA-Capital 2003
  - HBS prices 2008–2009 → ISA-Capital 2008
  - HBS prices 2008–2009 → ISA-Capital 2015
  In the case of the ISA-Capital 2015, there was absence of databases with prices registered in the same reference period. Thus, data on prices referring to the most recent survey available were adopted for pairing. Therefore, the food prices from the HBS-IBGE 2008-2009 were updated by using specific price deflators per food item for the municipality of São Paulo to maintain the relative prices for matching the ISA-Capital 2015.
2. Occurrence of similarity between the socioeconomic strata of the individuals interviewed in the HBS-IBGE in relation to the socioeconomic strata of the individuals interviewed in the ISA-Capital (10 strata of the household income per capita, considering only the monetary income) in order to express the differences in the food prices paid by the individuals according to the income level.
3. Occurrence of similarity in the household composition in terms of number of the family members and age groups of the residents (children under 7 years old, adolescents under 10–19 years old, and seniors under 60 years or older) in which they represent the economies of scale due to the household size and to identify the differences in the characteristics of products purchased according to the life stage with potential influence on the food prices.

The purpose of establishing the pairing criteria to link the price data from the HBS-IBGE with food consumption from the ISA-Capital was based on the need to ensure the similarity between the patterns of food consumption represented by the expenditures of the households from the HBS in relation to the individuals living in São Paulo municipality interviewed in the context of the ISA-Capital.

The data extracted from the HBS-IBGE encompassed the variables referring to the matching criteria (number of household members, age, household income per capita, municipality of residence, and year) and information on the food items purchased (type, weight, and expenditure).

The food expenditure data from the HBS-IBGE was converted into prices per gram (in case of food) or per milliliter (in case of beverages). In the case of multiple registries of acquisition of the same food item within the same matching group (households with identical pairing characteristics), we calculated the mean price per unit (grams or milliliters).

The information on food intake of the individuals interviewed in the ISA-Capital was obtained through application of the 24-hour dietary recall (24HR), which records the foods and beverages consumed in the period before the interview.

The procedures for data collection of the ISA-Capital, including the 24HR, are available in the publication of Thompson et al. (14), and followed the Multiple-Pass Method procedures comprising five sequential steps to increase the response rate and maintain the attention of the respondent: rapid listing of foods, list of forgotten foods, time and occasion of food consumption, detailing and review of the list, and final review (15).

## Results

The results comprise step-by-step examples of the application of the linkage method by using the data from the HBS-IBGE and the ISA-Capital. The use of the composition of variables adopted for pairing allowed data matching to follow one of the eight actions defined to minimize the possibility of the missing cases. It is important to highlight that action number #1, based on pairing of all the key variables (income, number of household residents, family characteristics, and food name), presented high correspondence between the datasets (94.4% in 2003, 92.6% in 2008, and 81.2% in 2015); thus, it represented the main strategy for the linkage in the study. All the key variables were used to match the prices from the HBS with food consumption of the individuals interviewed in the target survey (ISA-Capital) promoting pairing of the substantial proportion of cases (Table 1).

**Table 1:** Pairing characteristics and its corresponding actions in the linkage procedures of food prices from the Household Budget Survey-Brazilian Institute of Geography and Statistics (HBS-IBGE) to the Inquérito de Saúde de São Paulo (ISA-Capital).

**Information**						**Pairing procedure**		**% Of variables correspondence**		
**Household income**	**Household composition**				**Food name**	**#**	**Action**	**2003**	**2008**	**2015**
	**Number of residents**	**Children <7 years old**	**Adolescents**	**Older Adults**						
Yes	Yes	Yes (for all)			Yes	1	Consider variables in the pairing	94.37	92.6	81.17
Yes	Yes	No (at least one item)			Yes	2	Consider income and number of residents	0.00	0.00	0.86
Yes	No	Yes (for all)			Yes	3	Consider income and family profile	0.00	0.00	0.00
Yes	No	No (at least one item)			Yes	4	Consider income	0.00	0.00	0.00
No	Yes	Yes (for all)			Yes	5	Consider number of residents and family profile	5.42	7.22	16.30
No	Yes	No (at least one item)			Yes	6	Consider number of residents	0.00	0.00	1.61
No	No	Yes (for all)			Yes	7	Consider family profile	0.00	0.00	0.00
No	No	No (at least one item)			Yes	8	Consider only food name	0.21	0.18	0.06
*N*								2,398	1,662	1,742

*Source: The authors*.

On the other hand, the pairing procedure referring to action number #5 (i.e., use the number of household residents, family characteristics, and food for pairing data) was adopted for minor proportion of cases due to the absence of information on the household income per capita in the survey (missing data cases). There were only few cases of the individuals with missing data on all the key pairing variables (i.e., requiring the adoption of action #8, only the food description to perform pairing) corresponding to the proportion of <0.25% of the linkage process (0.21% in 2003, 0.18% in 2008, and 0.06% in 2015) (Table 1).

Following, the data organized in two datasets were connected by using the matching food codes according to the pairing of the household characteristics:

- Data on the food items consumed obtained from the 24HR of the ISA-Capital
- Data on the food purchases recorded in the HBS-IBGE including price per gram or milliliter

After matching the databases, the food prices from the HBS-IBGE were used to estimate the cost of foods consumed by individuals interviewed in the ISA-Capital allowing the estimation of costs per gram and costs per calorie of the diet and mean cost of food groups of interest in the analysis. It is important to highlight the difference between prices and costs in the context of the study.

It is important to highlight the difference between the prices and costs in the context of the study. Price represents the market value of goods, i.e., the amount paid by the consumers to purchase the product or service. On the other hand, cost represents the value of the resources required to produce the goods, i.e., the monetary value of the inputs of their production. Therefore, the estimation of the diet costs should be based on prices of the food items comprising preparations and meals.

One example refers to the preparations consumed by the individuals interviewed in the ISA-Capital such as the fruit smoothie: the prices of papaya, banana, and apple in the 2008–2009 HBS were 0.18317, 0.22023, and 0.26823 Brazilian reais per 100 g, respectively, while the cost of the fruit smoothie was 0.15501 Brazilian reais per 100 g (Table 2).

**Table 2:** Excerpt on the application of correction factors and cooking indexes to obtain the weight of ingredients, food items, and preparations ready-to-eat in the ISA-Capital.

**Meal**	**Correction factor (CF)**	**Cooking index (CI)**	**Purchase price (R$/g)**	**Edible price (R$/g)**	**%**	**Final price (R$/g of consumption)**
**Farofa**						
Ingredient 1 - Breadcrumbs	1.00	1.00	0.0029279	0.0029279	60	0.0017567
Ingredient 2 - Chicken egg	1.15	1.11	0.0038074	0.0036750	20	0.0007350
Ingredient 3 - Bacon	1.00	0.22	0.0435791	0.0095874	10	0.0009587
Ingredient 4 - Sausage	1.00	0.64	0.0063751	0.0040801	10	0.0004080
**Price of the meal**			0.0566895	0.0202703	100	0.0038585
**Stuffed eggplant lasagna**						
Ingredient 1 - Eggplant	1.08	0.85	0.0018617	0.0014652	50	0.0007326
Ingredient 2 - Prime ground meat	1.14	0.60	0.0110468	0.0058141	20	0.0011628
Ingredient 3 - Mozzarella cheese	1.00	1.00	0.0088449	0.0088449	15	0.0013267
Ingredient 4 - Potato	1.06	0.95	0.0013275	0.0011897	15	0.0001785
**Price of the meal**			0.0230809	0.0173140	100	0.0034006
**Fruit smoothie**						
Ingredient 1 - Papaya	1.39	1.00	0.0018317	0.0013178	40	0.0005271
Ingredient 2 - Banana	1.55	1.00	0.0022023	0.0014208	40	0.0005683
Ingredient 3 - Apple	1.18	1.00	0.0026823	0.0022731	20	0.0004546
**Price of the meal**			0.0067163	0.0050117	100	0.0015501

*Source: Own elaboration on the data from the Brazilian HBS corrected through the application of the correction factor (CF) and cooking index (CI) (16–18)*.

The next step referred to the estimation of prices per calorie by using the price per 100 g of the food item divided by the calories per 100 g, considering that the prices of food items for consumption at home usually refer to the whole raw food item:


$$\begin{array}{l} W_{f}=W_{i}\times \left(1-C_{f}\right) \end{array}$$

where *W*_*f*_ = final weight of the foods ready for consumption, *W*_*i*_ = initial weight of the whole raw foods, and *C*_*f*_ = correction factor. The correction factor refers to the ratio between the portion of edible food (excluding inedible parts) and/or cooking index (changes in the food weight due to physical, chemical, and biological cooking processes).

Appropriate correction factors and cooking indexes were applied to the food items consumed to obtain the price of food ready-to-eat according to the 24HR record (e.g., boiled, baked, fried, or grilled) by using indicators obtained in the literature (16–18).

The estimation of net food weight, excluding inedible parts, was based on the following equation:


$$\begin{array}{l} NW=\frac{CF}{GW} \end{array}$$

Where: *NW* = net weight or edible weight; *CF* = correction factor from the literature, and *GW* = gross weight or purchase weight.

The calculation of the cooked food weight was estimated through the following equation:


$$\begin{array}{l} CW=\frac{CI}{NW} \end{array}$$

Where: *CW* = final cooked weight, *CI* = cooking or conversion index from the literature, and *NW* = net weight or edible weight (Table 2).

In the case of food preparations (e.g., farofa, lasagna, or fruit smoothie), the ingredients required to prepare the dish were identified and their respective prices were used to estimate the cost of the dish according to their proportions in the recipe. For example, the cost of farofa was calculated based on the following proportions: 60% breadcrumbs, 20% chicken egg, 10% bacon, and 10% sausage (Table 2).

Another important issue in the linkage of variables measured in monetary units, such as prices, incomes, and expenditures, refers to the application of deflators required for updating monetary values throughout time in order to ensure comparability.

In this study, the construction of specific price deflators for each food item was based on the Broad Consumer Price Index of the Brazilian Institute of Geography and Statistics (IPCA-IBGE) for the metropolitan region of São Paulo resulting in the composition of deflators for 246 food items in the six reference periods to incorporate the price variations due to inflation.

Considering that the IBGE performs continuous price surveys throughout time in representative sample of retail stores, the availability of detailed deflators per food item in São Paulo municipality allowed to perform the updating process of the specific food items in the municipality during the period of the study, maintaining the relative food prices.

The adoption of specific deflators for each food item was important to maintain the relative prices from the periods of reference of the ISA-Capital, considering that the food items present diverse price variation trajectories throughout time. Therefore, maintaining relative prices allow the reproduction of market prices at the time of acquisition.

Examples of the deflators applied to update food prices and household income per capita in the study, calculated by using the IPCA-IBGE, are presented in the study (Tables 3, 4). The prices of the main food groups were updated to the reference period of July 2020, while the deflators of household income per capita were designed to update the monetary values to the reference period of the ISA-Capital (December 2015).

**Table 3:** Price deflators for the main food groups in the study to the reference period of July 2020, according to the date of the interviews of the individuals in the ISA-Capital.

**Reference period: July 2020**											
**Item**		**Date of interview**									
		March/03	April/03	May/03	June/03	July/03	August/03	September/03	October/03	November/03	December/03
1	Food and Beverage	3.0845	3.0264	2.9994	2.9709	2.9685	2.9885	2.9996	2.9696	2.9549	2.9513
11	Food at home	2.7935	2.7392	2.7110	2.6879	2.6928	2.7197	2.7375	2.7053	2.6983	2.6961
1101	Cereals, legumes and nuts	2.3411	2.3251	2.2945	2.0943	2.0693	2.1432	2.2050	2.1958	2.2346	2.2235
1103	Tubers, roots and vegetables	3.6824	3.0355	2.9320	3.2333	3.6095	4.0633	4.5861	4.3665	4.5141	4.2727
1104	Sugars and derivatives	1.9235	1.8986	1.8516	1.8163	1.8211	1.8579	1.8879	1.9000	1.9600	1.9959
1105	Vegetables	2.7546	2.8778	3.1393	3.5269	3.7552	3.8264	4.0397	3.9981	4.0639	4.0992
1106	Fruits	1.3041	1.2579	1.2808	1.4056	1.5112	1.5622	1.5604	1.5016	1.538	1.6501
1107	Meet	5.0383	5.0539	5.0575	5.0444	5.0948	5.1035	4.9795	4.7664	4.6625	4.6333
1108	Fish	3.0165	2.9872	2.7843	2.9317	3.0802	3.1982	3.2005	3.1194	3.1388	3.0542
1111	Milk and dairy products	2.9438	2.9207	2.8550	2.7471	2.6819	2.6630	2.6716	2.6758	2.6716	2.6847

*Source: Own elaboration on the data from the Broad Consumer Price Index (36)*.

**Table 4:** Deflators adopted for updating the household income per capita to the reference period of the ISA-Capital 2015 (December 2015) according to the date of the interviews of the individuals.

**Survey year**	**Date of interview**	**Deflator for per capita income**
2003	March/03	2.04565714
	April/03	2.02480168
	May/03	2.01312556
	June/03	2.00530487
	July/03	2.00831734
	August/03	2.00310926
	September/03	1.99831331
	October/03	1.98166730
	November/03	1.97751452
	December/03	1.97435555
2008	September/08	1.55902659
	October/08	1.55343423
	November/08	1.54493708
	December/08	1.54231514
	January/09	1.54216092
	February/09	1.53846860
	March/09	1.52853313
2015	September/14	1.13565523
	October/14	1.12832114
	November/14	1.12360201
	December/14	1.11801195
	January/15	1.11101257
	February/15	1.09448584
	March/15	1.08097367
	April/15	1.06699602
	May/15	1.06084313
	June/15	1.05357347
	July/15	1.04531548
	August/15	1.03712221
	September/15	1.03463908
	October/15	1.02734493
	November/15	1.01727392

*Source: Own elaboration on the data from the Broad Consumer Price Index (36)*.

In the case of specific foods without correspondence between the HBS and the ISA-Capital (e.g., seaweed, mussels, flaxseed), the prices were obtained through market research in three large market chains operating in the municipality of São Paulo. The mean prices obtained for each product were deflated to the reference period corresponding to the ISA-Capital through application of the specific food deflator from the IPCA.

Finally, considering the possibility of comparison on the diet costs across international studies, it is important to perform the conversion of the monetary values from the local currency unit (in the case of Brazil, Brazilian reais, R$) to the international currency unit in purchasing power parity (PPP), i.e., the International Monetary Units (IMUs) (2020). Thus, it is important to match the reference period of the PPP conversion factor with the reference period of prices, income, and expenditures; for instance, the monetary values of the Brazilian database and the PPP conversion factor should be presented for the same period (e.g., 2020).

In this study, the PPP conversion factor adopted referred to the PPP on private consumption [PPP conversion factor, private consumption (local currency unit (LCU) per international $)], obtained from the World Bank database for the conversion of monetary data into the monetary units comparable across the countries around the world (19), i.e., the values comparable independently of the local cost of living.

The linkage of datasets of the food prices from the HBS-IBGE and the food intake recorded in the ISA-Capital showed that the mean diet costs were $8.45 per person per day (*SD* = ±0.38) in 2003, $8.72 (*SD* = ±0.24) in 2008, and $9.62 (*SD* = ±0.23) in 2015. Diet costs increased during the period of analysis, especially among the individuals in the higher income quintile, and the individuals with children older than 7 years, adolescents, and older adults in the household (Table 5).

**Table 5:** Diet costs (mean and SD) according to the household characteristics of individuals interviewed in the ISA-Capital 2003-2015 (São Paulo, Brazil).

**Household characteristics**	2003^*^		2008^*^		2015^*^	
	**Mean**	**SD**	**Mean**	**SD**	**Mean**	**SD**
**Household income**						
1st quintile	5.46	0.26	8.46	0.75	8.74	0.46
2nd quintile	6.96	0.52	7.73	0.33	8.57	0.31
3rd quintile	7.39	0.29	8.12	0.50	8.69	0.33
4th quintil	9.07	0.50	9.28	0.40	10.13	0.42
5th quintile	11.93	1.08	9.91	0.48	11.67	0.64
**Household composition**						
**Household residents**						
1–2	8.63	0.71	10.32	0.66	9.19	0.28
3–5	9.13	0.53	7.83	0.27	9.83	0.27
≥6	7.65	0.33	8.76	0.30	9.67	0.66
**Children** ** <7 years old**						
No	8.19	0.32	8.22	0.54	9.17	0.38
Yes	8.97	0.75	8.90	0.25	9.74	0.24
**Adolescents**						
No	8.36	0.36	8.95	0.25	9.02	0.33
Yes	8.53	0.55	9.86	0.42	9.87	0.25
**Older Adults**						
No	8.25	0.38	8.72	0.27	9.53	0.25
Yes	8.81	0.53	9.66	0.58	9.78	0.34
**Total**	8.45	0.38	8.72	0.24	9.62	0.23

* *Values in the international currency unit in the purchasing power parity (PPP), e.g., the International Monetary Units (IMUs) (2020)*.

## Discussion

To the best of our knowledge, this is the first study to describe the details of the methodological procedures for the linkage of datasets on economic data with health and nutrition surveys, i.e., aggregation of databases with information on food prices and food consumption at individual level with population representativeness. Considering the importance of diet costs and food prices in the causal trajectory between socioeconomic level and diet quality, the lack of surveys encompassing data on food consumption and prices poses substantial barriers to the comparison of evidence on the costs associated with diet quality in diverse populations (1, 20, 21).

This study presented the methodology for the integration of data from independent sources through data pairing (match) based on the correspondence of target characteristics between individuals, households, or other research units, an alternative to the lack of integrated datasets including simultaneously food prices and food intake.

The linkage technique is usually adopted in the population studies out of the field of health economics; however, it may be adapted to enable economic evaluation studies through selection of key variables of interest and well-established matching criteria. The main advantages of the linkage refer to the potential use for detailed analysis of the health and nutrition surveys and promote the increase in quantity and quality of data available in the population-based studies (22, 23).

In addition, we described the steps required for the integration of databases from the different sources available in Brazil, presenting the best practices for establishment of the rigorous pairing criteria based on the similarity patterns between the key characteristics of individuals and households from the different surveys. In this study, the matching of the target variables (location, year, family income per capita, number of residents, and sociodemographic characteristics of household members) allowed the connection of 94.4% (2003), 92.6% (2008), and 81.2% (2015) of the records.

Considering the studies with more than one 24HR, estimation of the diet costs should be performed by using the mean food intake values registered in the records to establish the standard diet costs for comparison with the nutritional quality. The estimation of usual dietary intake may be performed through the removal of intrapersonal variance by using the statistical models proposed by the National Cancer Institute or the Multiple Source Method (24–26).

There is conflicting evidence on the association between the diet costs and quality; however, major part of the studies indicates that healthy dietary patterns usually present higher cost in comparison with less healthy diets, especially in developed countries (1, 2, 27, 28). Other studies indicate the possibility of healthy diets with lower cost depending on the method for the measurement of costs (prices per gram, per unit of energy, or per serving), socioeconomic level of the population, and geographical region considered in the study (5, 6, 29–32).

The use of the costs per unit of energy (calories) of food is usually recommended in the context of the public health studies, considering that the quality of the diet is the main focus of the analysis (5). The cost per calorie remains a suitable metric for the comparison of costs of diverse diets (5), being widely adopted in the literature on the field of nutrition and development economics.

The utilization of diet costs per mass (grams or liters) is usually indicated for the comparison of food items with similar nutritional characteristics in the context of the consumer choices, i.e., considering the price per weight of the edible portion for the comparison of foods marketed in the different formats (e.g., fruits or vegetables in natural or frozen) (5, 33). The adoption of correction factors and cooking indexes is important to ensure the proper assessment of costs of ready-to-eat items, removing inedible parts (e.g., skin, seeds, shell, bones). In addition, there is reduction or increase of the food weight depending on the cooking procedures (33–35). The diet costs per mass represent a useful metric to the health economics researchers, allowing to estimate the consumer demand models encompassing the variations in the amounts purchased of the same product, i.e., food products available for the purchase in the different forms (5, 33).

Regarding information on the monetary values, such as incomes, prices, and expenditures, there are important procedures that should be followed to ensure the comparability of data collected in the different periods. First, it is important to choose a single reference date to incorporate the inflation rates accumulated throughout time. Second, the relative prices should be maintained through utilization of the specific-item deflators in case of data collection on prices that are performed in different periods than the interview of the individuals (36).

Third, the use of the PPP-adjusted international currency units is essential to allow the direct comparisons of the monetary values across the countries (19, 31, 37). The employment of the PPP-adjusted estimates on the private consumption accounts for the differences in the exchange rates and purchasing power, eliminating divergences due to the income levels, standards of living, and the government incentives in the diverse countries.

Finally, the method adopted for the measurement of diet costs should reflect the market prices or, alternatively, the opportunity costs in the location and period of the survey. Therefore, it is essential to match the cases by pairing through key variables and, in addition, to control for confounding factors associated with the diet quality in the statistical analysis, e.g., sociodemographic (age, gender), economic (income), lifestyle (physical activity level, smoking), health indicators (body mass index, chronic diseases) (20).

The study presents certain limitations, especially referring to the missing data in the key variables to perform the matching (due to the absence of responses or failures in questionnaire coding). The occurrence of excessive cases of the missing data may compromise the validity of results. However, the implementation of pairing between the HBS-IBGE and the ISA-Capital showed high success rates, ensuring robustness of the results. Other limitation of the study refers to the potential differences between actual food prices paid by the individuals interviewed in the ISA-Capital and food prices recorded in the HBS-IBGE. Yet, the procedures that were adopted in the study minimize the potential deviations, particularly referring to the concept of opportunity costs (i.e., alternative utilization of the resources) in the estimation of diet costs.

Thus, it is important to highlight the strengths of the study. Considering the constraints imposed on studies referring to diet costs, investigations usually gather information through market research or standardized tables on the food prices without connection to the characteristics of the consumers. This study showed that the linkage comprises an alternative approach to obtain the food prices in the population-based surveys (such as the Brazilian HBS-IBGE) that may be paired with the health and nutrition surveys (such as the ISA-Capital) through information on location, period, and key pairing variables referring to the socioeconomic and demographic characteristics linked to the consumer behavior.

In addition, the pairing criteria were established a priori and the food-specific deflators were used to minimize the bias related to the missing data and provide proximity to the monetary values in the retail stores of the municipality, generating the estimates of reliable diet costs throughout the period of analysis. Finally, the use of databases with samples representative at the population level (the HBS-IBGE and the ISA-Capital) allows the inferences on diet costs in the municipality of São Paulo in the reference period of this study.

## Conclusion

This study showed the application of the linkage method for the estimation of diet costs and prices of food items and food groups of interest in the field of nutrition by pairing and merging information from the HBS-IBGE with the databases of health and nutrition surveys without information on prices. The linkage method may be an important resource for researchers in health economics and nutrition to perform studies on the association between diet quality and food demand and/or consumer behavior toward food choices at the population level. The results referring to diet costs and food prices and the presentation of methods for the estimation of direct diet costs may be used to support the design of public policies of health directed toward strategies for the promotion of healthy lifestyles based on the diet quality. In addition, the linkage procedures may be adopted to estimate diet costs in diverse countries with availability of datasets on household expenditures and health and nutrition surveys to generate evidence on the association between diet quality and food demand characteristics.

## Data Availability Statement

The original contributions presented in the study are included in the article/supplementary material, further inquiries can be directed to the corresponding author.

## Ethics Statement

The studies involving human participants were reviewed and approved by the Research Ethics Committee of the School of Public Health of the University of São Paulo (Reference Number: 11751019.3.0000.5421). The patients/participants provided their written informed consent to participate in the surveys.

## Author Contributions

All authors listed have made a substantial, direct and intellectual contribution to the work, and approved it for publication.

## Funding

This study was carried out with support from the São Paulo Municipal Health Department (Grant Number 2013-0.235.936-0); the State of São Paulo Research Foundation (Grant Numbers 98/14099-7, 2007/51488-2, 2009/15831-0, 2012/22113-9, 2017/05125-7); and the National Council for Scientific and Technological Development (Grant Numbers 502948/2003-5, 481176/2008-0, 473100/2009-6, 472873/2012-1, 402674/2016-2, 430850/2016-6, 301597/2017-0, 301109/2019-2). This study was financed in part by the Coordenação de Aperfeiçoamento de Pessoal de Nível Superior (CAPES)—Brasil (Financial Code 001). The funders had no role in the conception, collection, analysis, and interpretation of the data; in the writing of the article; and in the decision to submit it for the publication.

## Conflict of Interest

The authors declare that the research was conducted in the absence of any commercial or financial relationships that could be construed as a potential conflict of interest.

## Publisher's Note

All claims expressed in this article are solely those of the authors and do not necessarily represent those of their affiliated organizations, or those of the publisher, the editors and the reviewers. Any product that may be evaluated in this article, or claim that may be made by its manufacturer, is not guaranteed or endorsed by the publisher.
